# Investigating the status of pyrethroid resistance in UK populations of the cabbage stem flea beetle (*Psylliodes chrysocephala*)

**DOI:** 10.1016/j.cropro.2020.105316

**Published:** 2020-12

**Authors:** Caitlin E. Willis, Stephen P. Foster, Christoph T. Zimmer, Jan Elias, Xianmin Chang, Linda M. Field, Martin S. Williamson, T.G. Emyr Davies

**Affiliations:** a Biointeractions and Crop Protection Department, Rothamsted Research, West Common, Harpenden, UK; b Syngenta Crop Protection, Werk Stein, Schaffhauserstrasse, Stein CH4332, Switzerland; c Royal Agricultural University, Stroud Rd, Cirencester, Gloucestershire, UK

**Keywords:** Cabbage stem flea beetle: oilseed rape, Pyrethroid resistance

## Abstract

The cabbage stem flea beetle, *Psylliodes chrysocephala* L. is a major pest of winter oilseed rape in several European countries. Traditionally, neonicotinoid and pyrethroid insecticides have been widely used for control of *P. chrysocephala*, but in recent years, following the withdrawal of neonicotinoid insecticide seed treatments, control failures have occurred due to an over reliance on pyrethroids. In line with previous surveys, UK populations of *P. chrysocephala* were found to exhibit high levels of resistance to the pyrethroid lambda-cyhalothrin. This resistance was suppressed by pre-treatment with the cytochrome P450 inhibitor PBO under laboratory conditions, suggesting that the resistance has a strong metabolic component. The L1014F (kdr) mutation in the voltage-gated sodium channel, which confers relatively low levels (10–20 fold) of resistance to pyrethroids, was also found to be widespread across the UK regions sampled, whereas the L925I (s-kdr) mutation was much less common. The current survey also suggests that higher levels of pyrethroid resistance have spread to the North and West of England, and that resistance levels continue to remain high in the South East.

## Highlights

• UK populations of cabbage stem flea beetle exhibit high levels of resistance to pyrethroid insecticides.
• Pyrethroid resistance in UK populations is largely the result of increased metabolism.
• Resistance has advanced to the North and West of England.
• Resistance levels are highest in Yorkshire and the Humber and the South East of the UK.

## 1 Introduction

The cabbage stem flea beetle, *Psylliodes chrysocephala* (Coleoptera: Chrysomelidae) is an established and key insect pest of winter oilseed rape, particularly in the UK (Graham and Alford, 1981) and Germany (Zimmer et al., 2014), and is a significant pest of other *Brassica* species in several European countries (Bromand, 1990; Bartlet and Williams, 1991; Bartlet et al., 1996). *P. chrysocephala* inflicts damage at both the larval and adult stage, with the tunnelling of the larvae into the leaf petioles and main stems causing the most damage through weakening of the upper section of the roots and lower parts of the stems (Williams, 2004). When infestation is high, the plant tips distort, the stems wilt and the infested plants become more susceptible to fungal infections such as *Phoma lingam* (Alford, 2003), the bacterial disease *Erwinia* sp. and frost damage (Højland et al., 2015; Højland and Kristensen, 2018). Adult *P. chrysocephala* cause damage by feeding on stems, cotyledons and the first true leaves during crop emergence resulting in ‘shot-holing’ symptoms, leading to poor plant vigour or potential seedling death before emergence when fields are heavily infested (Williams, 2010). Prior to 2014, *P. chrysocephala* affected approximately 67% of the area of oilseed rape grown in the UK causing an annual 1% yield loss (Clarke et al., 2009). However, in 2014, serious crop losses due to adult beetles (2.7% of the national crop) were recorded, the most serious losses (5–14%) being in eastern and southern England (Wynn et al., 2014). In the autumn of 2015, a more extensive survey found that over 65% of crops had some damage, and that the damage was more widely distributed across the country than in 2014, although nationally only 1% of crops were lost (Alves et al., 2015). Subsequent surveys have confirmed that the average numbers of larvae per plant have risen substantially in all regions since 2014 (as summarised in Dewar, 2017).

Prior to December 2013, control of *P. chrysocephala* relied on the protection of oilseed rape seedlings by systemic neonicotinoid seed treatments containing either imidacloprid, thiamethoxam or clothianidin, followed by the application of foliar pyrethroid sprays later in the season if needed (Højland et al., 2015; Højland and Kristensen, 2018). However, in December 2013, the European regulatory authorities (EU Commission, 2013) banned the use of neonicotinoid seed treatments on oilseed rape, leading to the increase in *P. chrysocephala* and the increased use of pyrethroid sprays. Today, pyrethroids (e.g. lambda-cyhalothrin) are the only class of insecticide that remain for chemical control of *P. chrysocephala* in the UK and other parts of mainland Europe.

The continuous use of pyrethroids to control *P. chrysocephala*, coupled with the lack of alternative insecticides with different modes of action, has led to a high selection pressure, driving the development and spread of resistance. Resistance to pyrethroids was first reported in 2008, in north-western Mecklenburg, Western Pomerania, a major oilseed rape growing area in Northern Germany (Heimbach and Müller, 2013). Zimmer et al. (2014) reported the presence of the L1014F kdr mutation in the voltage-gated sodium channel, with high frequencies of the allele (90–100%) being found in populations collected from across Northern Germany, with the beetles exhibiting low level resistance against a range of pyrethroids including lambda-cyhalothrin. More recently, studies by Højland et al. (2015) and Højland and Kristensen (2018) have shown that pyrethroid resistance resulting from the kdr mutation is also present in populations from both Demark and the UK, whilst in Germany it has spread further south. Despite the presence of kdr in UK populations, Højland et al. (2015) found that the high pyrethroid resistance levels, with control failures being observed at the full field rate, did not completely correlate with the *kdr* genotype suggesting that another mechanism of resistance, such as metabolic resistance, is also present. Given the lack of alternative insecticides with different modes of action, the presence and spread of pyrethroid resistance is concerning for the chemical control of *P. chrysocephala*.

The present study has determined the current status, extent and geographical spread of pyrethroid resistance in UK populations of *P. chrysocephala*. Bioassays, based on glass vial exposure of adult beetles to lambda-cyhalothrin, were carried out on samples collected in 2018 and 2019 to examine how resistance had changed over this time across the UK. The presence of the *kdr* and super-*kdr* target-site mutations in UK populations was also monitored, and the potential contribution of a metabolic resistance component in the beetles assessed by pre-treatment with the synergist PBO, which is a cytochrome P450 inhibitor.

## 2 Methods

### 2.1 Collection of field samples of *Psylliodes chrysocephala*

In July/August 2018 and 2019, live *P. chrysocephala* adults were collected from oilseed rape pods freshly harvested from the fields at Rothamsted Research, Harpenden, Hertfordshire, using a hand-held battery-powered pooter. Insects were maintained at 15 ± 1 °C, with 65% relative humidity in a light:dark photoperiod of 12:12 h. Adults were kept in a mesh cage and fed continuously on a diet of Chinese cabbage *(Brassica rapa* spp*)*. Further samples were received by post from oilseed rape fields across the UK and were kept in sealed plastic bags or plastic containers containing Chinese cabbage or oilseed rape plant material and moist tissue paper, maintained in the same environmental conditions as the Rothamsted samples.

### 2.2 Bioassays to test the effect of pyrethroids on *Psylliodes chrysocephala*

*P. chrysocephala* samples were tested for resistance to the pyrethroid lambda-cyhalothrin using a glass vial bioassay based on IRAC (Insecticide Resistance Action Committee) Method 031 (www.irac-online.org/methods/weevils-and-flee-beetles/2014). Glass vials (14 ml: 7 cm tall/2 cm diameter) (S Murray and Co, Surrey, UK) were prepared by coating the inner surface with different concentrations of the insecticide. Initial stock solutions were prepared by diluting the technical grade insecticide in technical grade acetone. Three doses, equivalent to 4%, 20% and 100% of the recommended field application rate of lambda-cyhalothrin (7.5 g a.i./ha) were used. The controls were glass vials treated with acetone only. To coat vials, 500 μl of solution was pipetted into the vials which were then placed horizontally without lids on a roller in a fume hood. Vials were rotated at room temperature for at least 2 h until all the acetone had evaporated. Vials were then left vertically at 4 °C overnight before attaching the screw tops the following day.

The adult beetles (see 2.1) were used within a few days of collection and only those capable of walking or jumping when released onto a tray inside a three-sided Perspex cage were collected, using a hand-held battery-powered pooter. A minimum of ten beetles were transferred from the inverted pooter through a small funnel into each vial. The vials were then resealed and left at 18 ± 1 °C under a 16:8 h light:dark photoperiod. After 24 h, the beetles were transferred to untreated glass vials without lids under upturned 200 ml plastic disposable cups (VWR International Ltd, Dublin, Ireland), to allow for a potential recovery which can occur in insects with metabolic resistance. After a further 24 h, the beetles were released onto a tray and individuals scored using a fine paint brush according to three categories: ‘mobile’ (capable of jumping or walking in a coordinated way), ‘affected’ (incapable of jumping or coordinated movement) or ‘dead’ (no movement). Scoring of the beetles from each vial was done for 10 min to avoid adults that were simulating death, a behaviour shown by this species that has probably evolved through predation pressure. Results were expressed as percentage mortalities. Following scoring, beetles in each category were transferred to Eppendorf tubes and snap frozen using liquid nitrogen before being stored in a freezer at −80 °C.

### 2.3 TaqMan PCR assay to detect the presence of kdr/skdr in *Psylliodes chrysocephala*

TaqMan genotyping assays (Livak, 1999) were used to determine the presence of the mutations responsible for the kdr (L1014F) and super-kdr (L925I) sodium channel substitutions in individual adult beetles. Primer Express v.2.0 (Life Technologies) was used to design the primer and probe sequences for the assays (Table 1). In both assays, VIC reporter dye-labelled probes were used to detect the wild-type susceptible allele and 6-FAM reporter dye-labelled probes to detect the resistant allele. Each probe contained a 3’ non-fluorescent quencher dye.

**Table 1:** Primer and probe sequences used for TaqMan assays to detect the L1014F (kdr) and L925I (skdr) mutations in *Psylliodes chrysocephala*.

Primer/Probe		Sequence
Primers	kdr-F	GGACTGTATGCTAGTCGGTGATGT
	kdr-R	GCAAAGCCAAGAAGAGATTCAGTA
	skdr-F	GCCAAGTCATGGCCAACTT
	skdr-R	TATAATGCACAGCACAAAGGTCA
Probes	kdr-VIC	TTACCACAAGATTACC
	kdr-FAM	TTACCACAAAATTACC
	skdr-VIC	TGGGTGCTTTAGGTAA
	skdr-FAM	TGGGTGCTATAGGTAA

PCR reactions (15 μl) contained 1.5 μl (50 ng) genomic DNA, 7.5 μl SensiFast probe mix (Bioline Reagents Ltd, UK), 0.375 μl of kdr or skdr primer/probe mix (800 nM of each primer and 200 nM of each probe) and sterile water. Reactions were run on an Applied Biosystems 7900HT real-time PCR system, with initial incubations at 50 °C for 2 min and 95 °C for 10 min, followed by 40 cycles of 95 °C for 15 s and 60 °C for 45 s. The increase in VIC and 6-FAM reporter dye fluorescence was monitored in real time and an allelic discrimination analysis performed using the 7900HT Sequence Detection System software.

### 2.4 Use of a synergist to identify the presence of metabolic resistance in *Psylliodes chrysocephala*

Pre-treatment with the insecticide synergist Piperonyl butoxide (PBO), obtained from Sigma-Aldrich (Missouri, USA), was used to detect potential metabolic resistance mechanisms *in-vivo*. PBO was diluted in technical grade acetone to give an equivalent concentration of 0.011 mg cm^−2^ (Højland et al., 2015). This dose was chosen because it did not cause control mortality when tested. 500 μl of solution was then used to coat glass vials (see 2.2). Ten beetles per replicate were transferred to the PBO-coated vials for 1 h before being transferred to either untreated control vials or vials coated with lambda-cyhalothrin at the 100% field rate (7.5 g a.i./ha). The beetles were then bio-assayed in parallel to beetles from the same sample not pre-exposed to PBO.

## 3 Results and discussion

### 3.1 Survey of pyrethroid resistance in *Psylliodes chrysocephala* across the UK

To determine the current extent and geographical spread of resistance to pyrethroid insecticides in UK *P. chrysocephala* populations, and how this compares to previous reports (Højland et al., 2015), bioassays with lambda-cyhalothrin were conducted on adult beetle samples from Rothamsted Research's farm in Hertfordshire and oilseed rape fields located across England, Scotland and Wales. The bioassays allowed the samples to be categorised as being either completely susceptible, or to contain beetles that were 0–25%, 25–50%, 50–75%, 75–99% and 100% resistant, depending on the percentage of beetles per sample surviving treatment with 7.5 g a.i. ha^−1^ lambda-cyhalothrin. Although lambda-cyhalothrin is used as an exemplar in these studies, other pyrethroids also contribute to the selection pressure in *P. chrysocephala* populations across Europe. The bioassay is an approved test method (method 031) for determining resistance in *P. chrysocephala* (IRAC, 2104) and was used by Zimmer et al. (2014) to monitor the emergence and geographic spread of pyrethroid resistance in *P. chrysocephala* in Germany, by Højland et al. (2015) to determine the spread of pyrethroid resistance in Danish, British and German samples and most recently by Højland and Kristensen (2018) when investigating lambda-cyhalothrin resistance in Danish populations. Similar bioassays have also been used to monitor the spread of pyrethroid resistance in European populations of pollen beetle (*Brassicogethes aeneus*), another major pest of oilseed rape (Zimmer and Nauen, 2011; Slater et al., 2011; Nauen et al., 2012).

**Fig. 1:**
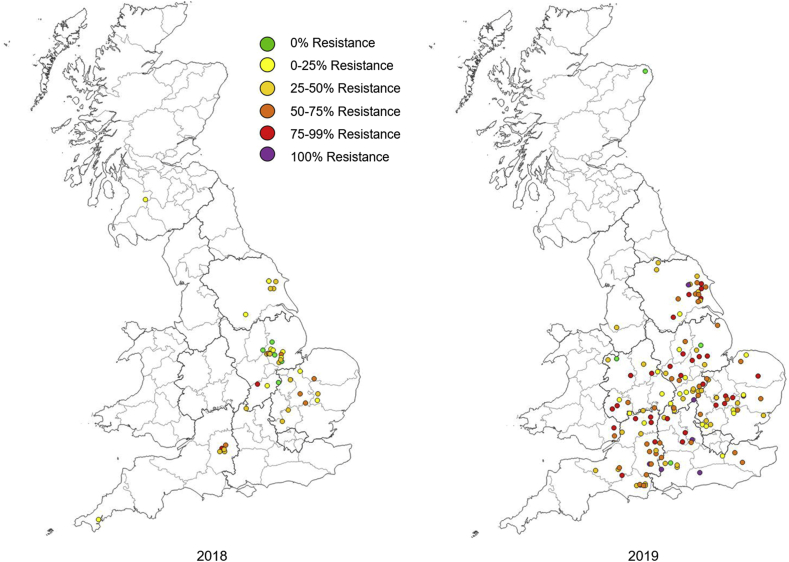
Pyrethroid resistance in *P. chrysocephala* in the UK for 2018 and 2019. The maps were created using QGIS (version 3.0.3) and use a 6-category colour scale to show the level of resistance. The map is divided into counties (light grey borders) and regions (dark grey borders).

In 2018, a total of 41 *P. chrysocephala* samples, obtained from four different regions across England, but primarily from counties in the East (Fig. 1), were tested. Of these only five samples were found to contain no mobile beetles at 100% of the recommended field rate for lambda-cyhalothrin, which would be expected if the sample was susceptible. However, for these five samples mortality was found to be <90% at 20% of the field rate, suggesting resistance is present as judged by the IRACs ‘susceptibility rating scheme’ (IRAC, 2104). The other 37 samples all showed some level of resistance with the highest resistance, at 89% being the sample from Bishop Cannings (Wiltshire).

In 2019 a total of 146 *P. chrysocephala* samples were obtained from across England, representing more of the country (Fig. 1), two samples were received from Wales and one from Scotland. Only the Scottish sample was found to be truly susceptible to lambda-cyhalothrin, displaying 100% mortality at 20% of the recommended field rate. Worryingly, several populations containing 100% resistant beetles were recorded for the first time in the UK. Overall, the distribution maps for pyrethroid resistance in UK populations of *P. chrysocephala* (Fig. 1) suggest that higher levels of resistance have spread to the North and West of England and that resistance levels continue to remain high in the South East.

**Fig. 2:**
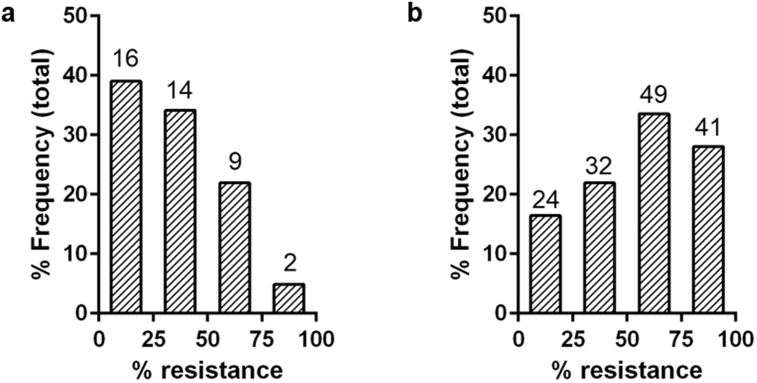
Histograms showing the shift in the relative % frequency of pyrethroid resistant *P. chrysocephala* in (a) 2018 and (b) 2019. Numbers show the raw count data.

Over the two years of monitoring, the percentage of highly pyrethroid-resistant beetles in the samples increased. The mean resistance level was significantly greater in 2019 (55.64%) compared to 2018 (32.9%) (two-sample *t*-test, t_185_ = −5.02, p < 0.001, SED = 4.529). Over the two years there was found to be a significant difference in the distribution of measurements across the resistance categories, *X*^*2*^(3) = 18.47, p < 0.001 (Fig. 2). In 2018 the percentage of beetles in the 0–25% resistance category was 39%, whereas in 2019 this decreased to 16%. In 2018 5% of samples were in the 75–100% resistance category whereas in 2019 this increased to 28%.

To assess whether there was any impact of the spatial variation over which the samples were collected, analysis of covariance was undertaken based on year, adjusting for the easting and northing coordinates as covariates. There was no evidence of a linear association between the covariates and the outcome at the 5% significance level. The analysis was then repeated with 3 geographically extreme data values omitted (two observations in Scotland and one in Cornwall). Again, there was no evidence of a linear association between the covariates and the outcome. However, in both cases the analysis showed a significant difference in mean resistance between the two years (p < 0.001). Scatter plots of the easting and northing coordinates plotted against resistance did not indicate any other non-linear association.

Further analysis was undertaken to assess regional and county-level differences for the 2019 data. The mean resistance levels (Table 2a) were higher than the national average in the South East (60.39%), South West (57.83%), and Yorkshire and the Humber (63.32%). South-East Wales had the highest mean resistance (72.50%) but only two samples were tested. Analysis of variance (ANOVA), incorporating a nested treatment structure to reflect counties nested within regions, showed that mean resistance levels did not differ significantly between regions (F_8,113_ = 1.28, p = 0.262). The standard errors of the differences between means (SEDs) at the regional level were also calculated (Table 2b). There was also found to be no significant difference in mean resistance levels between counties within the same region (F_24,113_ = 1.06, p = 0.395). The residual mean square from the ANOVA was 675.3. The absence of statistically different mean resistance levels suggests there are no resistance ‘hotspots’ and that resistance is highly localised, almost on a farm-by-farm basis.

**Table 2:** **(a)** Summary of average resistance levels by region and county and **(b)** Standard error of differences between means at the regional level.

A		
Region and County	Number of Samples	Average resistance level (%)
**East Midlands**	**29**	**51.28**
Leicestershire	7	60.43
Northamptonshire	12	48.58
Nottinghamshire	3	49.67
Lincolnshire, Parts of Kesteven	6	45.33
Lincolnshire, Parts of Lindsey	1	60
**East of England**	**29**	**53.72**
Bedfordshire	3	57.33
Cambridgeshire	8	72.25
Essex	4	31.5
Hertfordshire	3	26
Huntingdonshire	2	57
Norfolk	3	53.33
Suffolk	6	55
**North West**	**1**	**40**
Lancashire	1	40
**Scotland**	**1**	**0**
Aberdeenshire	1	0
**South East**	**18**	**60.39**
Berkshire	2	80
Hampshire	6	50.67
Kent	3	61.67
Oxfordshire	5	64
Surrey	1	18
Sussex	1	100
**South East Wales**	**2**	**72.5**
Monmouthshire	2	72.5
**South West**	**35**	**57.83**
Dorset	7	53.57
Gloucestershire	8	57.88
Somerset	4	57.5
Wiltshire	16	59.75
**West Midlands**	**12**	**48.33**
Herefordshire	4	60.25
Shropshire	3	46.67
Staffordshire	3	46.33
Warwickshire	1	10
Worcestershire	1	50
**Yorkshire and the Humber**	**19**	**63.32**
East Riding of Yorkshire	14	68.93
North Riding of Yorkshire	3	43.33
West Riding of Yorkshire	2	54
**Grand Total**	**146**	**55.64**

B										
Region		Standard Error of Differences								
East Midlands	1	*								
East of England	2	6.82	*							
North West	3	26.43	26.43	*						
Scotland	4	26.43	26.43	36.75	*					
South East	5	7.8	7.8	26.7	26.7	*				
South East Wales	6	19	19	31.83	31.83	19.37	*			
South West	7	6.53	6.53	26.36	26.36	7.54	18.89	*		
West Midlands	8	8.92	8.92	27.05	27.05	9.68	19.85	8.69	*	
Yorkshire and the Humber	9	7.67	7.67	26.66	26.66	8.55	19.32	7.41	9.58	* *
		1	2	3	4	5	6	7	8	9

### 3.2 Pyrethroid resistance mechanism(s) in *P. chrysocephala*

The TaqMan assays (see 2.3) were used to detect the presence of the L1014F (kdr) and L925I (s-kdr -like) substitutions in the 2018 and 2019 *P. chrysocephala* samples (Table 3). In 2018, 40 beetles from seven UK samples of *P. chrysocephala* were tested using only individuals that had survived the 100% field rate of lambda-cyhalothrin, enabling the genotype associated with the resistant, mobile phenotype to be determined. The samples were from Great Saxham (Suffolk), Bishop Cannings (Wiltshire), Rothamsted (Hertfordshire), Linton (Cambridgeshire), Feltwell (Norfolk) and Horbling and Grantham (Lincolnshire). The L1014F mutation was present at all sites, with 47.5% of the beetles being homozygous for the resistant allele (RR), 37.5% heterozygous (SR) and the remaining 15% *kdr* SS, although this genotype was not present in the Suffolk or Wiltshire populations. The detection of *kdr* SS genotypes in beetles that displayed the mobile phenotype after treatment with the label rate of lambda-cyhalothrin, strongly suggests the presence of another resistance mechanism in *P. chrysocephala.* We also identified *kdr* RR (homozygote) genotypes in beetles that did not survive lambda-cyhalothrin treatment, confirming that the L1014 mutation on its own is not able to confer protection to the field rate dose (results not shown). In contrast to L1014F, the L925I mutation, which is predicted (based on studies in other insects) to be associated with higher resistance levels to pyrethroids than kdr, was much less common, with two samples (Norfolk and Hertfordshire) showing only the SS genotype and the overall percentage of beetles showing the homozygous L925I genotype (RR) being only 2.5%. Of the six beetles homozygous for the susceptible *kdr* allele (SS), one also displayed the homozygous resistant s-*kdr* allele (RR) and two displayed the heterozygous resistant s-*kdr* allele (SR) (data not shown). As three beetles were susceptible for both the *kdr* and s-*kdr* allele this suggests the presence of another resistance mechanism. Direct sequencing of sodium channel fragments carrying the mutations showed that L1014F (kdr) and L925I (s-kdr) are mutually exclusive and have arisen independently in different sodium channel alleles, thus limiting the number of genotypic combinations possible within individual beetles.

**Table 3:** Detection of kdr/skdr alleles in *P. chrysocephala* using TaqMan assay.

Region	County	Populations	No°	SS	SR	RR	%RR
2018				*kdr status*			
East Midlands	Suffolk	1	4	0	3	1	25%
	Wiltshire	1	8	0	4	4	50%
	Lincolnshire	2	10	2	2	6	60%
East of England	Cambridgeshire	1	8	2	2	4	50%
	Norfolk	1	6	1	3	2	33%
	Hertfordshire	1	4	1	1	2	50%
	Total	7	40	6 (15%)	15 (37.5%)	19 (47.5%)	
				*skdr status*			
East Midlands	Suffolk	1	4	3	1	0	0%
	Wiltshire	1	8	5	3	0	0%
	Lincolnshire	2	10	9	1	0	0%
East of England	Cambridgeshire	1	8	5	2	1	13%
	Norfolk	1	6	6	0	0	0%
	Hertfordshire	1	4	4	0	0	0%
	Total	7	40	32 (80%)	7 (17.5%)	1 (2.5%)	
2019				*kdr status*			
East Midlands	Suffolk	1	4	0	1	3	75%
	Wiltshire	1	5	3	1	1	20%
	Lincolnshire	2	16	2	8	6	38%
East of England	Cambridgeshire	1	8	1	6	1	13%
	Norfolk	1	8	2	4	5	63%
	Hertfordshire	1	5	1	1	3	60%
SE England	Oxfordshire	1	19	7	4	8	42%
Scotland	Aberdeenshire	1	10	10	0	0	0%
	Total	9	75	26 (34.7%)	25 (33.3%)	27 (36%)	
				*skdr status*			
East Midlands	Suffolk	1	5	5	0	0	0%
	Wiltshire	1	3	1	1	1	33%
	Lincolnshire	2	16	11	5	0	0%
East of England	Cambridgeshire	1	8	5	3	0	0%
	Norfolk	1	8	7	1	0	0%
	Hertfordshire	1	6	4	1	1	17%
SE England	Oxfordshire	1	20	13	4	3	15%
Scotland	Aberdeenshire	1	10	10	0	0	0%

	Total	5	76	56 (73.7%)	15 (19.7%)	5 (6.6%)	

In 2019, *P. chrysocephala* individuals were screened for kdr from sites close to those sampled in 2018 (a sample from Oxfordshire was also included) and again, only beetles that survived the 100% field rate of lambda-cyhalothrin were tested. The L1014F mutation was present at all sites except the one from Scotland. The percentage of beetles homozygous for the *kdr* resistance allele (RR) increased in three of the samples, Suffolk, Norfolk and Hertfordshire but decreased overall from 47.5% to 36%. Given that the percentage of beetles resistant to lambda-cyhalothrin in each sample increased between 2018 and 2019, but there was an overall decrease in the homozygous and heterozygous L1014F mutation, this further suggests the presence of another resistance mechanism in *P. chrysocephala*. Whilst the L925I (s-kdr) mutation was less common than the L1014F (kdr) mutation, it was found to be present in the Wiltshire and Hertfordshire samples which contained only the wild-type metabolic genotype (SS) in 2018. In the Oxford sample 15% of the beetles tested for the s-kdr mutation were homozygous for the resistant allele (RR). Overall the percentage of beetles showing the homozygous genotype (RR) was 6.6%.

### 3.3 Bioassays of *P. chrysocephala* using lambda-cyhalothrin and the synergist PBO

The insecticide synergist piperonyl butoxide has been shown to inhibit both P450 monooxygenases and esterases, thereby acting as a tool for the identification of metabolic resistance in insect samples (Young et al., 2006). To investigate the lack of correlation between lambda-cyhalothrin resistance and *kdr* frequency, and to determine whether P450 monooxygenases (and/or esterases) may play a role in mediating pyrethroid resistance in UK *P. chrysocephala* populations, synergist bioassays with PBO pre-treatments were conducted on five *P. chrysocephala* samples.

**Fig. 3:**
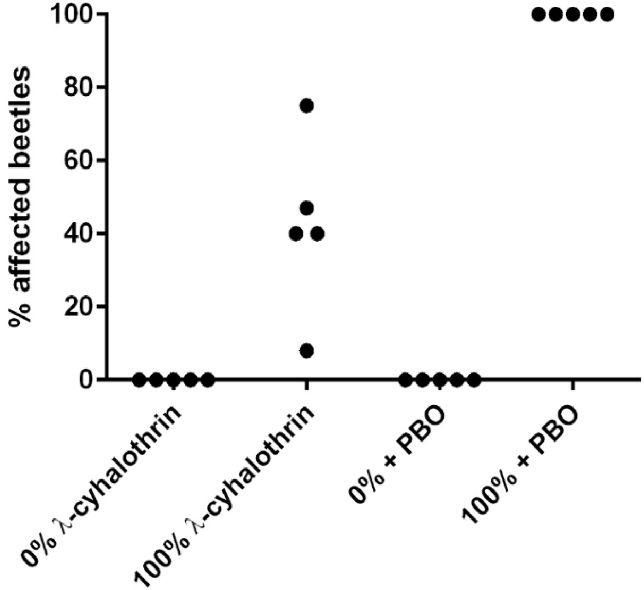
Restoration of insecticide (pyrethroid) susceptibility in *P. chrysocephala* following pre-treatment with PBO. Samples tested were from North Yorkshire, Wiltshire (x2), Leicestershire and Hertfordshire.

When exposed to lambda-cyhalothrin at the recommended field rate, the percentage of beetles affected was 47% (North Yorkshire), 75% (Wiltshire), 8% (Wiltshire), 40% (Leicestershire) and 40% (Hertfordshire) (Fig. 3). However, all adults pre-treated with PBO, prior to exposure to lambda-cyhalothrin at the same field rate were killed. This strongly suggests that a metabolic-based mechanism for pyrethroid resistance is present in *P. chrysocephala,* although it must be acknowledged that PBO has many more effects than just inhibiting enzymes, aiding cuticular penetration and increasing the insect's susceptibility to environmental stressors.

## 4 Conclusions

Since the EU-imposed ban on neonicotinoid seed treatments, pyrethroid insecticides have been widely used for chemical control of *P. chrysocephala* in the UK. This has resulted in a high selection pressure and led to the development of resistance, particularly in the South East of England. In the current study, populations of *P. chrysocephala* from around the UK were found to exhibit high levels of resistance to lambda-cyhalothrin, but this resistance was suppressed by the cytochrome P450 inhibitor PBO. This suggests that, as well as target site resistance, there may be P450 mediated-detoxification of lambda-cyhalothrin, although further research is required to identify the specific P450(s) involved and elucidate the exact mechanism of resistance. This resistance to pyrethroids has resulted in widely-reported control problems for this pest in the farming press (e.g. Clarke, 2014; Caswell, 2014; FarmingUK Team, 2015; Hill, 2017; FarmingUK Team, 2017; Case, 2018; Allison, 2019; Dyer, 2019; Gillbard, 2019) since the introduction of the neonicotinoid seed treatment ban.

Despite the development of resistance in *P. chrysocephala*, pyrethroids continue to be used on UK farms as there remains a lack of insecticides with alternative modes of action that can be deployed for resistance management. However, this continued reliance on pyrethroids is failing as a control strategy in many regions and is not sustainable in areas where resistance levels may appear low or non-existent. Since 2014, there has been a significant year by year decrease in the area of oilseed rape production in the UK, declining from 634,000 ha in 2014 (DEFRA, 2014) to 497,000 ha in 2019 (DEFRA, 2019). It is therefore particularly important that the extent and geographical spread of pyrethroid resistance in this pest continues to be monitored at a time when synthetic pesticides are becoming less favoured through EU legislation. Clearly there needs to be informed decision making on how to best deploy pesticides effectively in the future. Alternative strategies, such as the potential of the parasitoid *Microctonus brassicae* for biological control (Jordan et al., 2020), trap cropping (Barari et al., 2005) and the use of insect-resistant varieties of oilseed rape also offer options for *P. chrysocephala* control and are being further explored.

## CRediT authorship contribution statement

**Caitlin E. Willis:** Conceptualization, Methodology, Formal analysis, Investigation, Writing - original draft. **Stephen P. Foster:** Conceptualization, Methodology, Writing - review & editing, Supervision. **Christoph T. Zimmer:** Conceptualization, Methodology, Resources, Writing - review & editing, Supervision. **Jan Elias:** Resources, Writing - review & editing, Project administration. **Xianmin Chang:** Writing - review & editing, Supervision. **Linda M. Field:** Writing - review & editing, Supervision. **Martin S. Williamson:** Conceptualization, Methodology, Writing - review & editing, Supervision. **T.G. Emyr Davies:** Conceptualization, Methodology, Validation, Resources, Writing - review & editing, Supervision, Project administration, Funding acquisition.

## Declaration of competing interest

The authors declare that they have no known competing financial interests or personal relationships that could have appeared to influence the work reported in this paper.
